# Predictors of postoperative renal functional damage after nephron-sparing surgery

**DOI:** 10.1186/1477-7819-11-216

**Published:** 2013-08-29

**Authors:** Jun Qi, Yongjiang Yu, Tao Huang, Qiang Bai, Jian Kang, Junhao Liang, Yu Wu

**Affiliations:** 1 Department of Urology, Xinhua Hospital, School of Medicine, Shanghai JiaoTong University, No.1665 Kongjiang Road, Yangpu District, Shanghai, 200092, China

**Keywords:** Glomerular filtration rate, nephron-sparing surgery, renal tumor, predictors

## Abstract

**Background:**

Although nephron-sparing surgery has been reported not to affect total renal function, it is a non-negligible fact that functional damage of the operated kidney usually results, for various reasons. This study aimed to explore the effects of preoperative baseline characteristics, tumor characteristics, and function protection methods on postoperative renal damage.

**Methods:**

This study was a retrospective review of 51 patients who underwent open nephron-sparing surgery. The mean age of the patients (39 men, 12 women) was 54.2 ± 13.9 years, range 32 to 71 years. The glomerular filtration rate (GFR) was measured preoperatively and 6th months after the operation. Univariate analysis was used to screen indicators with significant differences in different levels of renal function damage. All variables found to be significant on univariate analysis were entered into a multiple logistic regression model to predict risk factors for renal function damage.

**Results:**

Univariate analysis showed that there was a significant difference in age, GFR of operated kidney, tumor diameter, tumor depth, and ischemic protection type between patients with little damage and those with heavy damage (*P* < 0.05). Forward stepwise logistic regression analysis suggested that age (odds ratio, 3.08; 95% confidence interval 1.78 to 7.04; *P* = 0.037), preoperative GFR of operated kidney (odds ratio, 0.51; 95% confidence interval 0.11 to 0.73; *P* = 0.033), and tumor diameter (odds ratio, 5.49; 95% confidence interval 2.14 to 7.88; *P* = 0.012) and depth (odds ratio, 5.82; 95% confidence interval 2.66 to 8.06; *P* = 0.010) were independent risk factors for postoperative renal function damage.

**Conclusions:**

Patients with older age, poor renal function, and large tumor diameter and depth might be at higher risk of renal function damage after nephron-sparing surgery.

## Background

Renal cell carcinoma, which accounts for 2% of solid tumors, is the most common urologic malignant tumor. It is estimated that renal cell carcinoma affects more than 40,000 patients annually in the United States and is responsible for approximately 13,000 deaths [1]. Radical nephrectomy has long been considered the most effective option for surgeons in the management of renal cell carcinoma. However, renal functional loss after radical nephrectomy contributes to the development of chronic kidney disease in the majority of patients, which is a significant risk factor for cardiovascular events and death [2].

Recently, nephron-sparing surgery has been the subject of much attention. Several clinical studies have already indicated that nephron-sparing surgery ensures favorable oncological results for tumors smaller than 4 cm compared with radical nephrectomy, in addition to preserving renal function [3-7]. Because of these beneficial outcomes, there has been an increase in the use of nephron-sparing surgery. At major urological institutions, nephron-sparing surgery is even largely used for tumors up to 7 cm in diameter, to extend elective indications [8-12]. However, it has been reported that nephron-sparing surgery impairs the function of the operated kidney because of temporary renal blood flow blockage for reduction of bleeding and loss of normal renal parenchyma around the tumor during resection and suture [13]. Therefore, assessing predictors of functional tissue damage of the involved kidney during nephron-sparing surgery to prevent it is still crucial for patients’ survival [14]. The aim of this study was to investigate preoperative indicators that might predict renal function damage in patients undergoing nephron-sparing surgery.

## Methods

### Patient selection

A total of 51 patients (approved by Xinhua Hospital, School of Medicine, Shanghai JiaoTong University ethics committee) who underwent open nephron-sparing surgery for a kidney tumor, as diagnosed by ultrasonography, computed tomography, or magnetic resonance imaging, were enrolled in this study between Jan 2009 and Dec 2011. The mean age of the patients (39 men, 12 women) was 54.2 ± 13.9 years, range 32 to 71 years. Patients with single kidneys were excluded from the study. Preoperative renal function was normal in all the patients. The patients’ blood levels of serum creatinine and urea nitrogen were 89 ± 20.1 μmol/l (range 78.5 to 109 μmol/l) and 5.7 ± 3.3 mmol/l (range 4.9 to 9.4 mmol/l), respectively. Preoperative backache was found in three cases; there was no gross hematuria or abdominal mass. All the tumors involved were unilateral (28 tumors on the left and 23 on the right kidney). According to the preoperative findings evaluated by computed tomography or magnetic resonance imaging, tumors were divided into large (diameter > 4 cm, 15 cases), medium (1 cm < diameter ≤ 4 cm, 28 cases) and small (diameter ≤ 1 cm, 8 cases) by their size. The tumors were also divided into exophytic (> 50% of the tumor circumference outside of renal parenchyma, 12 cases), central (≈ 50% of the tumor circumference outside of renal parenchyma, 26 cases) and intraparenchymal (< 50% of the tumor circumference outside of renal parenchyma, 13 cases) according to the depth of the tumor.

### Surgical technique

The patients were placed in a lateral decubitus position on the unaffected side and given general anesthesia. Using an extraperitoneal flank incision through the 12th rib, the renal pedicle was dissected free between the perirenal fascia and the major psoas muscle to allow for clamping by different vessel blockage types. Selective clamping of the artery, or blocking artery and vein, followed, depending on the distribution of the renal pedicle and the morphology and location of the renal mass [15]. Routinely, the renal artery was dissected free and clamped using a Rumel tourniquet (continuous artery blocking in 32 patients). Where patients had obvious tumor adhesions to the renal pedicle or larger tumor size, the renal pedicle, including the renal artery and renal vein, were clamped with a Rumel tourniquet (19 patients) to avoid vascular injury when isolating the renal artery. Owing to the difference in the personal habits of operators, different ischemic protection types were also used for operative warm ischemia of the kidney, including local cooling (30 patients), where sterile ice slush was placed around the kidney following vascular occlusion, and mannitol application (21 patients), where 250 ml 20% mannitol solution was given intravenously and rapidly 5 min before vessel occlusion. The perirenal fascia was opened immediately after the occlusion of blood vessels. The tumor was located after dissociation of the adipose capsule and should have been completely resected, leaving a 5 to 10 mm margin of normal renal parenchyma around the tumor. If there was damage to the collection system, it was oversewn with 3–0 absorbable sutures. The wound was closed in layers with 2–0 absorbable sutures in a figure-of-eight fashion. The vascular occlusion time should be less than 30 min before blood flow recovery. Drainage of retroperitoneal space was performed for 24 to 48 h and the incision was closed routinely. Patients were placed in a horizontal position postoperatively for 72 h.

### Assessment of renal function

Glomerular filtration rate (GFR) was measured using ^99m^Tc-diethylenetriamine pentaacetic acid dynamic renal scintigraphy preoperatively and at the postoperative 6th month. The level of renal function damage was divided into ‘no damage’ (ΔGFR ≤ 10 ml/min 1.73 m^2^), ‘slight’ (10 ml/min 1.73 m^2^ < ΔGFR ≤ 20 ml/min 1.73 m^2^), ‘moderate’ (20 ml/min 1.73 m^2^ < ΔGFR ≤ 30 ml/min 1.73 m^2^), and ‘serious’ (ΔGFR > 30 ml/min 1.73 m^2^), according to the difference between preoperative and postoperative GFR (ΔGFR) of tumor-involved kidney. The levels of no and slight renal function damage were further combined into ‘little’, and the levels of moderate and serious renal function damage were combined into ‘heavy’.

### Statistical analysis

One-way analysis of variance (ANOVA, for continuous variables) or chi-square test (for categorical variables) were used to filter indicators with significant differences in each level of renal function according to preoperative baseline characteristics (age and sex), tumor characteristics (location, maximum diameter, depth, and pathology) and function protection types (vessel blockage type, warm ischemic time, and ischemic protection type). All variables significant on univariate analysis were entered into a multiple logistic regression model to predict the risk factors for renal function damage (‘little’ or ‘heavy’). Statistical analyses were performed using SPSS 19.0 software. *P* < 0.05 was considered statistically significant.

## Results

Preoperative baseline characteristics are detailed in Table 1. All the patients successfully completed the surgery, and showed no serious complications. The protection types of renal function in surgery are shown in Table 2. Compared with preoperative GFR levels of the tumor-involved kidney, we found no, slight, moderate, and serious damage of renal function in 17, 19, 9, and 6 patients at the postoperative 6th month, respectively (Table 3). Univariate analysis indicated that there was a significant difference in age, preoperative GFR of tumor-involved kidney, tumor diameter, tumor depth, or ischemic protection type between different renal function damage groups (*P* < 0.05, Table 4). Forward stepwise logistic regression analysis suggested that age, preoperative GFR of tumor-involved kidney, tumor diameter and depth were independent predictors of postoperative renal function damage (*P* < 0.05, Table 5).

**Table 1:** Preoperative baseline characteristics of patients and tumors

**Parameters**	**Value**
Patients	
Sex (*n*)	
Male	39
Female	12
Age (mean ± standard deviation, years)	54.2 ± 13.9
Tumors	
Location (*n*)	
Left	28
Right	23
Diameter (mean ± standard deviation, cm)	3.1 ± 1.8
Maximum diameter (*n*)	
Φ ≤ 1 cm	8
1 cm < Φ ≤ 4 cm	28
Φ > 4 cm	15
Depth (*n*)	
Exophytic (> 50% of the tumor circumference outside of renal parenchyma)	12
Central (≈ 50% of the tumor circumference outside of renal parenchyma)	26
Intraparenchymal (< 50% of the tumor circumference outside of renal parenchyma)	13
GFR of tumor-involved kidney (mean ± standard deviation, ml/min)	45.9 ± 9.3
Pathology (*n)*	
Malignant	40
Benign	11

**Table 2:** Protection types of renal function in surgery

**Parameter**	**Value**
Vessel blockage type (*n*)	
Continuous artery blocking	32
Continuous artery and vein blocking	19
Ischemic protection type (*n*)	
Local cooling	30
Mannitol application	21
Warm ischemic time (*n*)	
≤ 25 minutes	40
> 25 minutes	11

**Table 3:** Function damage level according to ΔGFR in 51 patients

**Level of function damage ( *n *)**	**Value**
Little	
None	17
(ΔGFR ≤ 10 ml/min 1.73 m^2^)	
Slight	19
(10 ml/min 1.73 m^2^ < ΔGFR ≤ 20 ml/min 1.73 m^2^)	
Heavy	
Moderate	9
(20 ml/min 1.73 m^2^ < ΔGFR ≤ 30 ml/min 1.73 m^2^)	
Serious	6
(ΔGFR > 30 ml/min 1.73 m^2^)	

**Table 4:** Univariate analysis of baseline characteristics, tumor characteristics, and function protection types in each level of renal function

	**Little damage (*n *= 36)**	**Heavy damage (*n *= 15)**	***P***
**Baseline characteristics**			
Sex (*n*)			0.733*
Male	28	11	
Female	8	4	
Age (mean ± SD)	50.4 ± 6.9	56.4 ± 8.0	0.042^**^
Tumor location (*n*)			0.088*
Left	17	11	
Right	19	4	
Pathology (*n*)			0.860*
Malignant	28	12	
Benign	8	3	
GFR of tumor-involved kidney (ml/min 1.73 m^2^)	48.1 ± 8.8	41.5 ± 9.7	0.039^**^
**Tumor characteristics**			
Maximum diameter (*n*)			0.047*
Φ ≤ 1 cm	7	1	
1 cm < Φ ≤ 4 cm	22	6	
Φ > 4 cm	7	8	
Depth (*n*)			0.001*
Exophytic	10	2	
Central	22	4	
Intraparenchymal	4	9	
**Function protection types**			
Vessel blockage type (*n*)			0.794*
Continuous artery blocking	23	9	
Continuous artery and vein blocking	13	6	
Ischemic protection type (*n*)			0.017*
Local cooling	25	5	
Mannitol application	11	10	
Warm ischemic time			0.187^*^
≤25 minutes	30	10	
>25 minutes	6	5	

*Chi-square test, ** ANOVA. Italic *p* values indicate statistical significance.

**Table 5:** Forward stepwise logistic regression analysis of the factors on renal function damage

**Parameters**	**Heavy damage**	
	**Odds ratio (95% confidence interval)**	***P***
Age (years)	3.08 (1.78-7.04)	0.037
Baseline GFR (ml/min•1.73 m^2^)	0.51 (0.11-0.73)	*0.033*
Maximum diameter (cm)	5.49 (2.14-7.88)	*0.012*
Tumor depth (tumor position relative to the renal parenchyma)	5.82 (2.66-8.06)	*0.010*
Local cooling application	0.78 (0.58-1.51)	0.061

Italic *P* values indicate statistical significance.

## Discussion

The GFR is considered the best parameter for assessing renal function because it is directly proportional to the number of functioning nephrons [16]. In this study, we also assessed kidney function changes preoperatively and postoperatively by measuring GFR with ^99m^Tc-diethylenetriamine pentaacetic acid renal scintigraphy [17]. In the normal population, an irreversible decline of renal function occurs with aging, showing reduced GFR levels [18]. These changes are minor, but tend to be more obvious when the kidney suffers a trauma. Tolerance to surgery is poor if the kidney has preoperative dysfunction, and postoperative damage of kidney is often serious [19]. These were also confirmed in our study; preoperative GFR of tumor-involved kidney and age of patients were independent predictors of postoperative renal function damage.

Given the validity of GFR for assessing renal function, we divided patients into those with little and heavy damage, according to GFR changes, which we used to explore risk factors for renal function damage by comparing preoperative baseline characteristics, tumor characteristics, and function protection methods between two groups. The results indicated that tumor diameter, tumor depth, or ischemic protection methods were independent predictors of postoperative renal function damage.

Tumor size is the key factor in determining surgical procedures. Generally, nephron-sparing surgery is considered the optimum treatment for tumors smaller than 4 cm in diameter [20], and nephron-sparing surgery can be offered for tumors 4 to 7 cm in diameter, but the operation is very difficult [21]. When the tumor is larger than 7 cm in diameter, radical nephrectomy should be chosen because satellite nodules could exist in the periphery of the tumor, and lead to a high postoperative local recurrence rate [22]. In this study, 15 patients had tumors larger than 4 cm in diameter, and the maximum diameter was 4.8 cm. Our results showed that the risk of renal function damage after the operation increased as the tumor diameter increased. This may be because resection of a larger tumor volume requires a longer vascular occlusion time and causes a reduced residual normal renal parenchyma.

Traditionally, excision of the tumor with a 1 cm margin of normal-appearing parenchyma is a standard technique during nephron-sparing surgery, to avoid local recurrence [23]. However, margins of 10 mm may not be desirable, as they may result in the resection of tumors close to the renal hilum and result in increasing injury to the urinary collecting system and hilar vessels [24]. Thus, a peritumoral margin smaller than 10 mm is advocated (0.5 to 1.0 cm in our study) [25]. This kind of margin is easily achievable for exophytic tumors but not intraparenchymal or juxtahilar tumors, in which adjacent vascular structures or collecting systems are located [26]. In practice, there may be some deviations in the peritumoral margin (possible larger than 1 cm) after advancing the renal parenchyma at the marked margin for patients with intraparenchymal tumors, leading to renal damage. Our results also demonstrated this conclusion that patients with a deeper tumor location had a high risk of postoperative renal function damage, although local recurrence was not found in all follow-up patients.

Recent clinical studies demonstrated that warm ischemia should be within 20 to 25 min. When the warm ischemic time is ≥ 25 minutes, irreversible diffuse damage may be seen in surgically preserved nephrons [13,27,28]. Moreover, some scholars suggest zero ischemia partial nephrectomy only with preoperative superselective arterial embolization [29-33]. However, our results indicated there was no significant difference in ischemic time between different renal function damage groups. This may be associated with abundant collateral circulation from the adrenal and capsular branches, which leads to enhanced tolerance to ischemia [34].

Renal dysfunction is also related to the method of occlusion. Local cooling is the best way to protect renal function if the expected warm ischemic time exceeds 30 minutes [35]. Renal hypothermia in the range of 5 to 15°C is considered optimal for renal protection, based on the classic Ward experiment [36]. Nevertheless, it is difficult to fall to this standard as the kidney is exposed in the surgical field. For clinical practicability, a temperature of 20 to 25°C appears to provide complete renal protection for up to 3 h of arterial occlusion [37,38]. Univariate analysis of our results showed that local cooling benefited the renal function; however, multivariate analysis found no significant difference. This may be due to the short vascular occlusion time in our study, with a maximum time of 38 min.

## Conclusions

It is necessary to assess the preoperative risk and degree of renal function damage in order to protect residual renal function after nephron-sparing surgery. For patients with preoperative high-risk factors such as older age, poor preoperative renal function, large tumor size, or deep tumor location, such precautions as vascular occlusion and ischemia protection should be taken. Monitoring of patients with high-risk factors should be performed, to minimize renal function damage after nephron-sparing surgery.

## Abbreviations

ANOVA: Analysis of variance; GFR: Glomerular filtration rate.

## Competing interests

The authors declare that they have no competing interests.

## Authors’ contributions

JQ, YY, and TH designed the study. QB and JK carried out the study and interpreted the results. JL and YW wrote the manuscript. All authors read and approved the final manuscript.

## References

[B1] Jemal A Siegel R Ward E Murray T Xu J Thun MJ Cancer statistics, 2007 CA Cancer J Clin 2007 11 43 66 10.3322/canjclin.57.1.43 17237035

[B2] Lane BR Abouassaly R Gao T Weight CJ Hernandez AV Larson BT Kaouk JH Gill IS Campbell SC Active treatment of localized renal tumors may not impact overall survival in patients aged 75 years or older Cancer 2010 11 3119 3126 10.1002/cncr.25184 20564627

[B3] Uzzo RG Novick AC Nephron sparing surgery for renal tumors: indications, techniques and outcomes J Urol 2001 11 6 18 10.1016/S0022-5347(05)66066-1 11435813

[B4] Pahernik S Roos F Hampel C Gillitzer R Melchior SW Thuroff JW Nephron sparing surgery for renal cell carcinoma with normal contralateral kidney: 25 years of experience J Urol 2006 11 2027 2031 10.1016/S0022-5347(06)00271-0 16697793

[B5] Lau WK Blute ML Weaver AL Torres VE Zincke H Matched comparison of radical nephrectomy vs nephron-sparing surgery in patients with unilateral renal cell carcinoma and a normal contralateral kidney Mayo Clin Proc 2000 11 1236 1242 10.4065/75.12.1236 11126830

[B6] Lee CT Katz J Shi W Thaler HT Reuter VE Russo P Surgical management of renal tumors 4 cm. or less in a contemporary cohort J Urol 2000 11 730 736 10.1016/S0022-5347(05)67793-2 10687966

[B7] Lerner SE Hawkins CA Blute ML Grabner A Wollan PC Eickholt JT Zincke H Disease outcome in patients with low stage renal cell carcinoma treated with nephron sparing or radical surgery J Urol 1996 11 1868 1873 10.1016/S0022-5347(01)66032-4 8618276

[B8] Becker F Siemer S Rotering J Suttmann H Stockle M Nephron-sparing surgery Urologe A 2008 11 215 222 quiz 223 10.1007/s00120-008-1651-3 18250999

[B9] Nemr E Azar G Fakih F Chalouhy E Moukarzel M Sarkis P Khoury R Ayoub N Merhej S Partial nephrectomy for renal cancers larger than 4 cm Prog Urol 2007 11 810 814 10.1016/S1166-7087(07)92297-6 17633991

[B10] Mitchell RE Gilbert SM Murphy AM Olsson CA Benson MC McKiernan JM Partial nephrectomy and radical nephrectomy offer similar cancer outcomes in renal cortical tumors 4 cm or larger Urology 2006 11 260 264 10.1016/j.urology.2005.08.057 16461075

[B11] Dash A Vickers AJ Schachter LR Bach AM Snyder ME Russo P Comparison of outcomes in elective partial vs radical nephrectomy for clear cell renal cell carcinoma of 4–7 cm BJU Int 2006 11 939 945 10.1111/j.1464-410X.2006.06060.x 16643474

[B12] Becker F Siemer S Hack M Humke U Ziegler M Stockle M Excellent long-term cancer control with elective nephron-sparing surgery for selected renal cell carcinomas measuring more than 4 cm Eur Urol 2006 11 1058 1063 discussion 1063–1054 10.1016/j.eururo.2006.03.003 16630686

[B13] Funahashi Y Hattori R Yamamoto T Kamihira O Kato K Gotoh M Ischemic renal damage after nephron-sparing surgery in patients with normal contralateral kidney Eur Urol 2009 11 209 216 10.1016/j.eururo.2008.07.048 18706758

[B14] Nativ O Levi A Farfara R Halachmi S Moskovitz B Degree and predictors of functional loss of the operated kidney following nephron-sparing surgery: assessment by quantitative SPECT of 99m Tc-dimercaptosuccinic acid scintigraphy Advances Urol 2011 10.1155/2011/961525 PMC3154483 21845188

[B15] Cáceres F Núñez-Mora C Cabrera P García-Mediero J García-Tello A Angulo J Laparoscopic partial nephrectomy Actas Urol Esp 2011 11 487 493 10.1016/j.acuroe.2011.03.013 21641090

[B16] Zimmerman RE Mitchell K Davis RT Treves ST Calculation of glomerular filtration rate Pediatric Nuclear Medicine/PET 2007 3 New York: Springer 307 311

[B17] Choi JD Park JW Choi JY Kim HS Jeong BC Jeon SS Lee HM Choi HY Seo SI Renal damage caused by warm ischaemia during laparoscopic and robot-assisted partial nephrectomy: an assessment using Tc 99m-DTPA glomerular filtration rate Eur Urol 2010 11 900 905 10.1016/j.eururo.2010.08.044 21414862

[B18] Imai E Horio M Yamagata K Iseki K Hara S Ura N Kiyohara Y Makino H Hishida A Matsuo S Slower decline of glomerular filtration rate in the Japanese general population: a longitudinal 10-year follow-up study Hypertension Res 2008 11 433 441 10.1291/hypres.31.433 18497462

[B19] Mooney JF Ranasinghe I Chow CK Perkovic V Barzi F Zoungas S Holzmann MJ Welten GM Biancari F Wu VC Tan TC Cass A Hillis GS Preoperative estimates of glomerular filtration rate as predictors of outcome after surgery: a systematic review and meta-analysis Anesthesiology 2013 11 809 824 10.1097/ALN.0b013e318287b72c 23377223

[B20] Ljungberg B Hanbury DC Kuczyk MA Merseburger AS Mulders PF Patard JJ Sinescu IC Renal cell carcinoma guideline Eur Urol 2007 11 1502 1510 10.1016/j.eururo.2007.03.035 17408850

[B21] Peycelon M Hupertan V Comperat E Renard-Penna R Vaessen C Conort P Bitker MO Chartier-Kastler E Richard F Roupret M Long-term outcomes after nephron sparing surgery for renal cell carcinoma larger than 4 cm J Urol 2009 11 35 41 19012929 10.1016/j.juro.2008.09.025

[B22] Brookman-May S Johannsen M May M Hoschke B Gunschera J Wieland WF Burger M Difference between clinical and pathologic renal tumor size, correlation with survival, and implications for patient counseling regarding nephron-sparing surgery AJR Am J Roentgenol 2011 11 1137 1145 10.2214/AJR.11.6534 22021506

[B23] Novick AC Streem SB Walsh PC, Retik AB, Stamey TA, Vaughan J Surgery of the kidney Campbell’s Urology 1998 7 Philadelphia, PA: WB Saunders 2973 3061

[B24] X-s C Z-t Z Du J Bi X-c Sun G Yao X Optimal surgical margin in nephron-sparing surgery for T1b renal cell carcinoma Urology 2012 11 836 839 10.1016/j.urology.2011.11.023 22305422

[B25] Li Q-L Guan H-W Zhang Q-P Zhang L-Z Wang F-P Liu Y-J Optimal margin in nephron-sparing surgery for renal cell carcinoma 4 cm or less Eur Urol 2003 11 448 451 10.1016/S0302-2838(03)00310-5 14499679

[B26] Russo P Commentary on: prospective study of safety margins in partial nephrectomy: intraoperative assessment and contribution of frozen section analysis: Timsit MO, Bazin JP, Thiounn N, Fontaine E, Chretien Y, Dufour B, Mejean A, Department of Urology, Hôpital Necker-Enfants Malades, Paris, France, Urology 2006, 67 923–926 Urol Oncol 2006 11 559 560 10.1016/j.urolonc.2006.08.005 16635521

[B27] Lane BR Gill IS Fergany AF Larson BT Campbell SC Limited warm ischemia during elective partial nephrectomy has only a marginal impact on renal functional outcomes J Urol 2011 11 1598 1603 10.1016/j.juro.2010.12.046 21419452

[B28] Funahashi Y Hattori R Yamamoto T Sassa N Fujita T Gotoh M Effect of warm ischemia on renal function during partial nephrectomy: assessment with new ^99m^Tc-mercaptoacetyltriglycine scintigraphy parameter Urology 2012 11 160 164 10.1016/j.urology.2011.08.071 22070892

[B29] Gill IS Eisenberg MS Aron M Berger A Ukimura O Patil MB Campese V Thangathurai D Desai MM ‘Zero ischemia’ partial nephrectomy: novel laparoscopic and robotic technique Eur Urol 2011 11 128 134 10.1016/j.eururo.2010.10.002 20971550

[B30] Gill IS Patil MB de Castro Abreu AL Ng C Cai J Berger A Eisenberg MS Nakamoto M Ukimura O Goh AC Zero ischemia anatomical partial nephrectomy: a novel approach J Urol 2012 11 807 814 10.1016/j.juro.2011.10.146 22248519

[B31] Simone G Papalia R Guaglianone S Gallucci M ‘Zero ischaemia’, sutureless laparoscopic partial nephrectomy for renal tumours with a low nephrometry score BJU Int 2012 11 124 130 10.1111/j.1464-410X.2011.10782.x 22177008

[B32] Simone G Papalia R Guaglianone S Carpanese L Gallucci M Zero ischemia laparoscopic partial nephrectomy after superselective transarterial tumor embolization for tumors with moderate nephrometry score: long-term results of a single-center experience J Endourol 2011 11 1443 1446 10.1089/end.2010.0684 21797763

[B33] Simone G Papalia R Guaglianone S Forestiere E Gallucci M Preoperative superselective transarterial embolization in laparoscopic partial nephrectomy: technique, oncologic, and functional outcomes J Endourol 2009 11 1473 1478 10.1089/end.2009.0334 19694532

[B34] Lopera JE Suri R Kroma G Gadani S Dolmatch B Traumatic occlusion and dissection of the main renal artery: endovascular treatment J Vascul Int Radiol 2011 11 1570 1574 10.1016/j.jvir.2011.08.002 21937239

[B35] Secin FP Importance and limits of ischemia in renal partial surgery: experimental and clinical research Advances Urol 2008 10.1155/2008/102461 PMC2467455 18645616

[B36] Ward JP Determination of the optimum temperature for regional renal hypothermia during temporary renal ischaemia Bri J Urol 1975 11 17 24 10.1111/j.1464-410X.1975.tb03913.x 236801

[B37] Becker F Van Poppel H Hakenberg OW Stief C Gill I Guazzoni G Montorsi F Russo P Stöckle M Assessing the impact of ischaemia time during partial nephrectomy Eur Urol 2009 11 625 635 10.1016/j.eururo.2009.07.016 19656615

[B38] Novick A Renal hypothermia: *in vivo* and *ex vivo* Urologic Clin North Am 1983 11 637 644 6356550

